# Silk-Based Therapeutics Targeting *Pseudomonas aeruginosa*

**DOI:** 10.3390/jfb10030041

**Published:** 2019-09-13

**Authors:** Tina B. McKay, Rachael N. Parker, Morgan J. Hawker, Meghan McGill, David L. Kaplan

**Affiliations:** Department of Biomedical Engineering, Tufts University, 4 Colby St, Medford, MA 02155, USA; tmckay333@gmail.com (T.B.M.); rachael.parker@tufts.edu (R.N.P.); mrgnhawker@gmail.com (M.J.H.); meghan.mcgill@tufts.edu (M.M.)

**Keywords:** silk, *Pseudomonas aeruginosa*, scaffold, antibiotic

## Abstract

*Pseudomonas aeruginosa* (*P. aeruginosa*) infections may lead to severe damage of the cornea, mucosa, and skin. The highly aggressive nature of *P. aeruginosa* and the rise in multi-drug resistance, particularly in nosocomial settings, lead to an increased risk for permanent tissue damage and potentially death. Thus, a growing need exists to develop alternative treatments to reduce both the occurrence of bacterial infection and biofilm development, as well as pathological progression post-infection. Silk derived from *Bombyx mori* silkworms serves as a unique biomaterial that is biocompatible with low immunogenicity and high versatility, and thereby ideal for stabilizing therapeutics. In this study, we assessed the cytotoxicity of *P. aeruginosa* on human corneal stromal stem cells and two mucosal cell lines (Caco-2 and HT29-MTX). To determine whether antibiotic-immobilized scaffolds can serve as alternative therapeutics to free, diffuse forms, we developed novel gentamicin-conjugated silk films as functional scaffolds and compared antimicrobial effects and free gentamicin. The advantages of generating a surface coating with a covalently-bound antibiotic may reduce potential side-effects associated with free gentamicin, as well as limit the diffusion of the drug. Our results suggest that gentamicin conjugated to native silk and carboxyl-enriched silk inhibits *P. aeruginosa* growth. Development of stabilized antibiotic treatments with surface toxicity selective against bacteria may serve as an alternative approach to treat active infections, as well as potential prophylactic use as coatings in high-risk cases, such as post-surgical complications or prolonged hospitalization.

## 1. Introduction

*Pseudomonas aeruginosa* (*P. aeruginosa*) infections can affect a number of tissues, including the cornea, skin, gastrointestinal, urinary, and respiratory tracts [1]. The Centers for Disease Control and Prevention estimates that 51,000 cases of nosocomial infections are attributed to *P. aeruginosa* each year in the United States, with growing reports of multi-drug resistant strains leading to over 400 deaths per year [2]. Furthermore, chronic *P. aeruginosa* infections of the lung remain a major cause of high morbidity and mortality in cystic fibrosis patients, owing primarily to the high adaptability of the pathogen in vivo [3,4]. Thus, the growing risks of infection associated with post-surgical complications or immune-compromised conditions necessitate the development of novel therapeutic systems to reduce the growth or spreading of *P. aeruginosa* in healthcare settings.

The most common modes of transmission of a pathogen to a susceptible patient are mediated via droplet exposure, direct contact between a carrier and patient, or interaction with contaminated surfaces [5]. The rationale for our study focuses on developing novel therapeutics with effective antimicrobial surface activity as potential prophylactic coatings to reduce transmission of *P. aeruginosa*. We hypothesized that conjugating the effective antibiotic, gentamicin, via covalent bonding to silk polymers will enable surface immobilization of the drug, while maintaining bactericidal effects against *P. aeruginosa*. The dominant mechanism of action of free gentamicin involves inhibition of the bacterial 30S ribosome, thus inhibiting protein synthesis and leading to bacterial cell death [6]. Previous studies have also shown that gentamicin exhibits surface toxicity against *P. aeruginosa*, independent of cytosolic uptake with the formation of blebs within the bacterial membrane visible by electron microscopy at regions containing conjugated-antibiotic accumulation [7]. Further studies involving immobilization of gentamicin on the surface of nanoparticles or polymers have shown that conjugating gentamicin to substrates, including gold nanoparticles [8,9], polyethylene glycol hydrogels [10], and polypropylene polymers [11], results in the stabilization of the antibiotic with retained antimicrobial activity. Thus, surface toxicity of gentamicin may contribute to the total bactericidal effects in addition to 30S ribosomal inhibition, albeit with higher drug concentrations required to inhibit bacterial growth with minimum inhibitory concentrations (MIC) of 22 μM and 4 μM for gentamicin-conjugates and free gentamicin, respectively [7,12,13]. This approach has also been applied in the conjugation of other antibiotics with surface toxicity, including vancomycin [14,15,16] and antimicrobial peptides [17,18,19].

We propose a novel approach to conjugate gentamicin as surface coatings utilizing the biocompatible silk-based polymers derived from *Bombyx mori* silkworms [20]. Silk protein provides a useful biomaterial for stabilizing therapeutics owing to its low immunogenicity and chemical and structural adaptability [21]. Silk is composed of high abundances of glycine (G) (43%), alanine (A) (30%), and serine (S) (12%), with short repeats of GAGAGS, GAGAGY, and GAGAGVGY [22]. In biomaterial applications, silk is a versatile protein that can used to generate hydrogels, films, or sponges [23]. For ocular applications, silk films have been shown to maintain transparency with a low immune response upon corneal implantation in vivo, suggesting good biocompatibility with minimal disruption of normal vision [24]. Furthermore, modification of silk films as cell-surface substrates to include micropatterning or porosity is able to promote cell binding and alignment of human corneal fibroblasts [25] and corneal stromal stem cells (hCSSCs) [26], with the retention of the high expression of extracellular matrix proteins, including collagen types I and V. The application of silk biomaterials as controlled vehicles for the release of drugs or antibiotics using bulk-loaded films and hydrogels has previously been reported by our group [20,27,28]. Our current study investigated the acute response to bacterial inoculation on the timeline of 1–6 h to determine if the cell type contributed to susceptibility to *P. aeruginosa* toxicity. Furthermore, we studied the antimicrobial effects of gentamicin, both conjugated and blended forms, to determine if surface toxicity of gentamicin was sufficient to protect against *P. aeruginosa*-induced cell loss of stromal and mucosal cultures as a measure of therapeutic potential for prophylactic use in high-risk cases of infection. The goal of this study was to develop an effective silk-based antibacterial system with high biocompatibility using covalently-bound gentamicin to reduce issues associated with free gentamicin, including toxicity [29,30], frequent topical drug application or infusion [31], and an increased risk for the development of drug-resistance [32].

## 2. Results and Discussion

### 2.1. Design and Characterization of Gentamicin-Conjugated Silk Films

**Figure 1:**
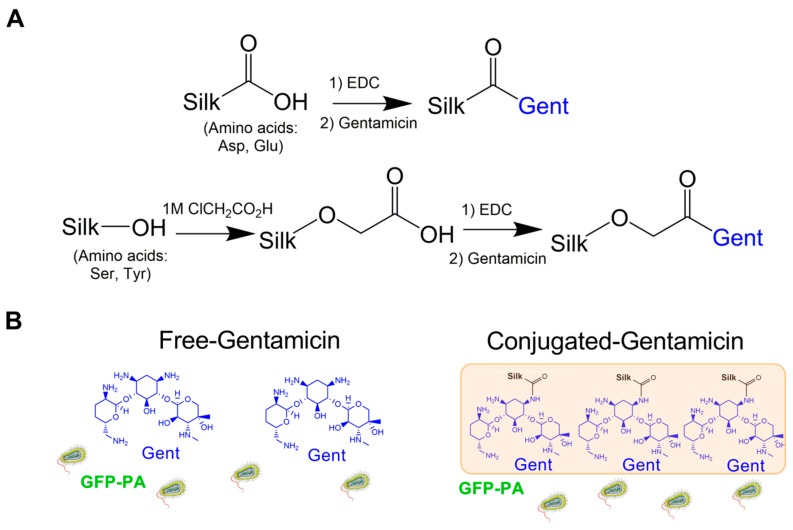
Development of gentamicin-conjugated silk scaffolds. (**A**) Reaction schemes showing 1-ethyl-3-(-3-dimethylaminopropyl) carbodiimide hydrochloride (EDC)-mediated coupling of native silk to form a covalent bond between gentamicin and aspartic acid and glutamic acid residues, and carboxyl-enrichment mediated followed by EDC coupling of gentamicin to free reactive carboxylic groups; (**B**) Proposed application of gentamicin-silk films in the treatment of bacterial infection by *P. aeruginosa*. Free-gentamicin applied to conditioned media results in dispersed drug availability with bactericidal effects mediated primarily via ribosomal inhibition. Conjugated silk–gentamicin films immobilize gentamicin to the surface of the scaffold, thereby providing a consistent antimicrobial surface. Bacteria pictorial was obtained from Servier Medical Art available at https://smart.servier.com based on a Creative Commons Attribution 3.0 Unported License.

Gentamicin contains four primary amines that are conducive to carbodiimide-mediated coupling to carboxylic acids. However, aspartic acid and glutamic acid are relatively low in abundance in the heavy chain of silk fibroin, collectively <2% of the total amino acids. To enrich the silk biomaterial with carboxylic acid moieties, we utilized chloroacetic acid to convert alcohol functional groups on the silk backbone (serine, tyrosine, and threonine (minor) residues, collectively ~18% of the total amino acids) to carboxylic acids groups [33,34] (Figure 1A). Previous studies have shown maintenance of gentamicin-bioactivity following conjugation to polymers or nanoparticles [8,11]. To assess the antimicrobial effectiveness of silk–gentamicin conjugates in our system, we performed EDC-mediated coupling of gentamicin to native silk and carboxyl-enriched silk to generate conjugated films with enriched surface gentamicin availability to compare to antimicrobial effects of free gentamicin forms (Figure 1A). We hypothesized that covalently-bound gentamicin to silk scaffolds will maintain antimicrobial activity, consistent with the surface bactericidal effects of gentamicin [7], in addition to 30S ribosomal inhibition [6], thus serving as a useful small molecule therapeutic agent for applications in both blended and conjugated forms (Figure 1B).

**Figure 2:**
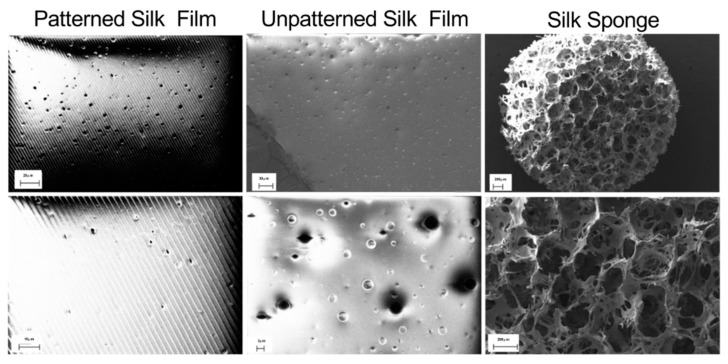
Topographical characterization of silk scaffolds using scanning electron microscopy (SEM). Polyethylene oxide (PEO)-generated pores (3–5 μM diameter) are evident on both patterned and unpatterned silk films. The porous silk sponge shows large cavities generated using salt-leaching (500–600 μM salt-particle diameter).

We performed topographical characterization of the silk scaffolds by scanning electron microscopy (SEM). Micropatterned silk films contained grooves roughly 2.5 μM apart with heterogenous distribution of pores generated using polyethylene oxide (PEO)-leaching of 3–5 μM diameter (Figure 2). Likewise, non-patterned porous silk films exhibited flat surfaces with both large (3–5 μM) and small diameter pores (<1 μM). The presence of micropores in the film may permit air- and liquid-perfusion, depending on the applied surface. Salt-leached silk sponges exhibited large caverns of roughly 200 μM diameter, consistent with a highly porous and absorbent material. These results validate and highlight the structural versatility of the silk scaffolds depending on the processing approach utilized.

**Figure 3:**
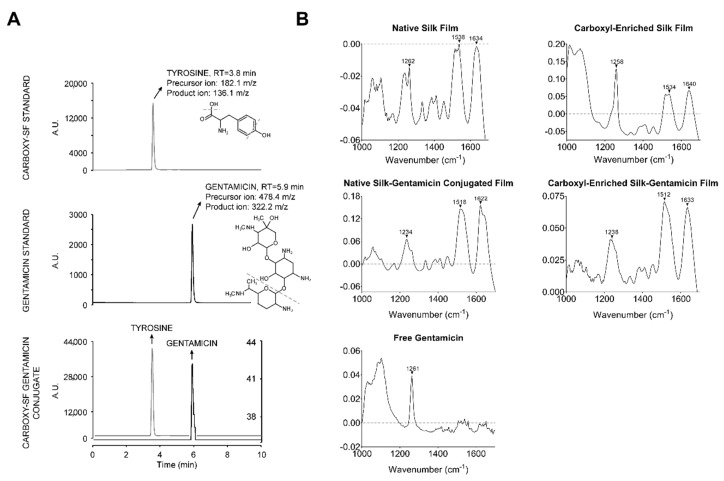
Liquid chromatography-tandem mass spectrometry (LC-MS/MS) and infrared (IR) spectra of silk controls and gentamicin-loaded silk scaffolds. (**A**) Confirmation of elution times and fragmentation patterns of the tyrosine ion in carboxyl-enriched silk standard (top) and gentamicin ion in gentamicin standard (middle); the presence of both ions in carboxyl-enriched silk gentamicin conjugate films confirms successful conjugation (bottom); (**B**) IR spectra of silk and gentamicin-loaded silk films and relevant controls. Carboxyl-enriched silk was cast onto a polydimethylsiloxane (PDMS) mold and analyzed by IR spectra.

To verify successful gentamicin-conjugation on bactericidal effects against *P. aeruginosa*, we validated EDC-mediated coupling of gentamicin to silk via liquid chromatography-tandem mass spectrometry (LC-MS/MS), following acid hydrolysis of silk and gentamicin standards and gentamicin-conjugated silk film samples (Figure 3A). We confirmed effective silk hydrolysis based on the presence of tyrosine in the silk standard (precursor ion 182.1 m/z, product ion 136.1 m/z, retention time 3.8 min), which was consistent with previous reports [35]. A standard solution of gentamicin showed a precursor ion at 478.4 m/z, product ion at 322.2 m/z, and a retention time of 5.9 min, consistent with literature reports [36]. Gentamicin-conjugated silk films contained both tyrosine and gentamicin peaks, confirming the success of the conjugation method. Further studies are required to determine stability of the silk–gentamicin bond in physiological environments.

Further characterization of silk films was performed using Fourier transform-infrared spectroscopy (FT-IR) (Figure 3B). Free gentamicin has no endogenous amide bonds, however, conjugation to silk leads to at least one amide bond between the free amine of gentamicin and the carboxylated serines and tyrosines of silk. Identification of differences between free gentamicin and bound gentamicin was not apparent by FT-IR because of the diverse functional groups found in the silk protein (Figure 3B). Using UV/visible spectroscopy, we validated that the high transparency of gentamicin-blended native silk films was maintained (>85%) within the visible light region (400–800 nm), supporting the use of these scaffolds in ocular applications requiring high transparency, such as in the case of prophylactic application to contact lenses to inhibit corneal infection (Supplemental Figure S1).

### 2.2. Efficacy of Gentamicin-Loaded Silk Biomaterials Against Bacterial Infection

*P. aeruginosa* (PA) grows as a rod-shaped bacterium on lysogeny broth (LB) agar and emits green fluorescence when transfected with green fluorescent protein (GFP) (Supplemental Figure S2A). The linear GFP expression of the strain utilized in our study (GFP-labelled *P. aeruginosa* (GFP-PA), ATCC 10455) has been reported to be correlative with bacterial growth up to 10^10^ colony forming units/mL [37], thus we assessed GFP expression as a measure of bacterial growth over time. We found that adherence of GFP-PA to decellularized corneal tissue was evident by t = 48 h post-inoculation, with clustering of bacteria and high protein accumulation consistent with biofilm formation, as shown by Sypro^®^ Ruby staining, a dye that binds exogenous protein (Supplemental Figure S2B).

**Figure 4:**
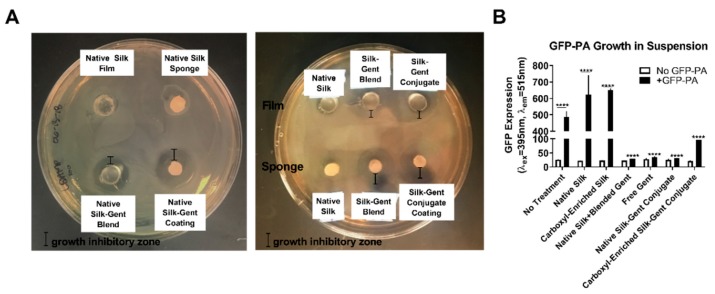
Bactericidal effects of gentamicin-loaded silk biomaterials. (**A**) Growth inhibitory assay using lysogeny broth (LB) agar containing 100 μg/mL ampicillin and inoculation with green fluorescent protein GFP-PA. Small discs of silk scaffold controls (silk films or sponges) or gentamicin-loaded scaffolds (blended, coated, or conjugated) were applied to the surface on a PDMS mold with inhibitory zone of bacterial growth visualized under brightfield at t = 24 h post-inoculation; (**B**) Quantification of total planktonic GFP-PA growth in epithelial media assessed spectroscopically based on GFP total fluorescence at t = 6 h post-inoculation with different treatments. Soluble solutions (native silk, carboxyl-enriched silk, carboxyl-enriched silk-gentamicin conjugate, and free gentamicin) and films (native silk + blended gentamicin and native silk–gentamicin conjugate) at concentrations of 1% w/v silk were tested to determine effectiveness at inhibiting bacterial growth. n = 3. Error bars represent standard error of the mean. **** *p* ≤ 0.0001 based on a two-way analysis of variance (ANOVA).

To assess the bacterial inhibitory zone of gentamicin–silk scaffolds on solid substrates, we applied small films on PDMS molds or silk sponges to the surface of an LB agar plate inoculated with a lawn of GFP-PA followed by incubation for t = 24 h (Figure 4A). In the absence of water-annealing, silk films readily dissolve in aqueous environments [38,39], allowing the release of silk and silk–gentamicin conjugated products into the surrounding agar. We found little antibacterial effects of native silk protein on bacterial growth, but found that gentamicin-loaded samples exhibited large inhibitory zones with comparable effects between gentamicin-blended and gentamicin-conjugated silk films and sponges (Figure 4A).

To determine the bactericidal effects of gentamicin-loaded biomaterials in solution, we performed a bacterial inhibition assay with silk and silk-gentamicin solutions, as well as with gentamicin alone to determine total GFP-PA growth in the absence or presence of the antimicrobial (Figure 4B). By 6 h post-inoculation, we found significant amounts of GFP-PA in the no treatment control group and silk controls with little GFP-PA detected in gentamicin-loaded solutions (blends and conjugates) (Figure 4B). A 1.3-fold increase in GFP-PA was detected in the carboxyl-enriched silk control, though not statistically significant (*p* = 0.065), suggesting that the presence of the carboxyl-enriched silk protein in solution may slightly increase bacterial growth independent of the antibiotic (Figure 4B). Treatment groups showed effective reduction in GFP-PA levels by 15-fold in the native silk–gentamicin conjugated films, comparable to the bactericidal effects of free gentamicin (14-fold) (*p* ≤ 0.0001, Figure 4B). Similarly, carboxyl-enriched gentamicin conjugates showed a significant reduction in bacterial growth up to 5-fold (*p* ≤ 0.0001), with the moderate reduction in bactericidal effects likely affected by the slight increase in bacterial growth in the presence of the carboxyl-enriched scaffold alone.

**Figure 5:**
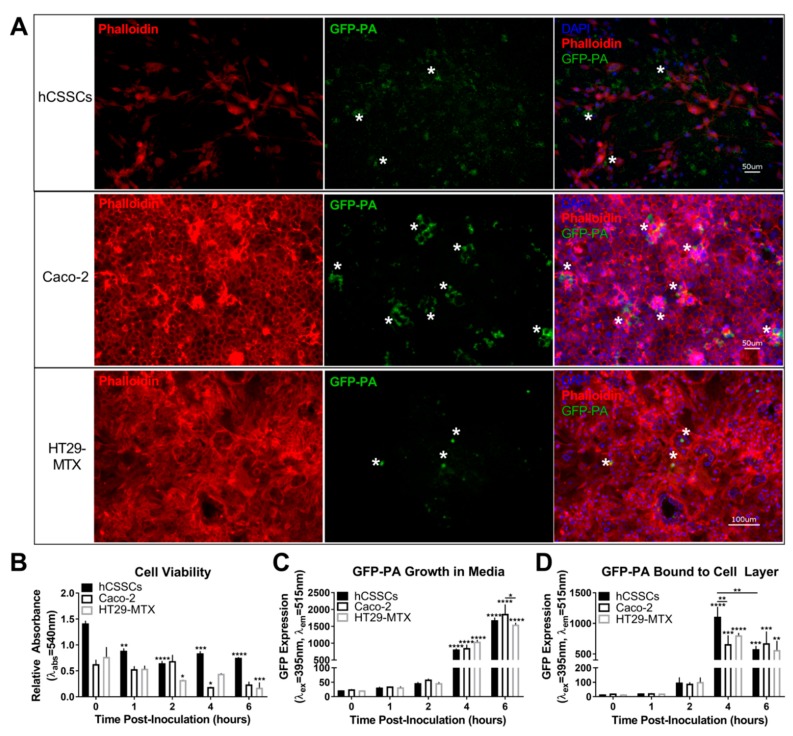
Effects of GFP-labelled *P. aeruginosa* (GFP-PA) in stromal (hCSSCs) and mucosal (Caco-2 and HT29-MTX) cultures. (**A**) Representative immunohistochemistry (IHC) images showing GFP-PA growth at t = 4 and 6 h for mucosal cells and hCSSCs, respectively. The asterisks (*) highlight regions of high GFP-localization on the surface of select human cells; (**B**) Cell viability assessed using an MTT (3-[4,5-dimethylthiazole-2-yl]-2,5-diphenyltetrazolium bromide) assay; (**C**) Abundance of planktonic; and (**D**) adherent GFP-labelled *P. aeruginosa* (GFP-PA) determined spectroscopically based on the GFP signal recovered from conditioned media or cell layer, respectively. n = 3 for each cell type. Error bars represent standard error of the mean. * *p* ≤ 0.05, ** *p* ≤ 0.01, *** *p* ≤ 0.001, **** *p* ≤ 0.0001 based on a one-way ANOVA with Dunnett’s multiple comparisons test.

We investigated the susceptibility of stromal (hCSSCs) and mucosal cell types (Caco-2 and HT29-MTX) to *P. aeruginosa* adhesion and cytotoxicity in order to establish a baseline for evaluating therapeutic effectiveness. We inoculated cultures of hCSSCs, Caco-2, and HT29-MTX with GFP-PA and analyzed for adhesion of the bacteria at t = 4–6 h post-inoculation (Figure 5A).

We found that localization of GFP-PA was proximal to the cell bodies of hCSSCs and non-stratified cells of Caco-2 cultures, suggesting that cell–cell contacts may be important in influencing the adhesion of GFP-PA to the surface (Figure 5A). Furthermore, we identified that Caco-2 cells that were loosely bound at the apical surface attracted GFP-PA adhesion, as can be noted with the localization of bacteria at these sites. Consistent with the presence of a mucus layer [40,41], HT29-MTX cells exhibited less confinement of GFP-PA to specific sites, but rather appeared dispersed above the cell layer.

To evaluate the cytotoxicity of *P. aeruginosa* in the context of the corneal stroma and mucosal cell types in vitro, we quantified cell loss via an MTT (3-[4,5-dimethylthiazole-2-yl]-2,5-diphenyltetrazolium bromide) assay. On the basis of the cell number, total hCSSC density was significantly reduced by 1 h post-infection (Figure 5B). Both Caco-2 and HT29-MTX cells showed increased resistance to *P. aeruginosa*-induced cell loss, with considerable retention of total cell number up to 2–4 h post-inoculation (Figure 5B). Interestingly, while total bacterial growth increased from 1 to 6 h in the conditioned media by 68-fold (*p* ≤ 0.0001), adhesion of bacteria was dependent on cell type (Figure 5C,D). hCSSCs showed the highest adhesion of GFP-PA by 4 h, consistent with significant cell loss (*p* ≤ 0.0001, Figure 5D).

**Figure 6:**
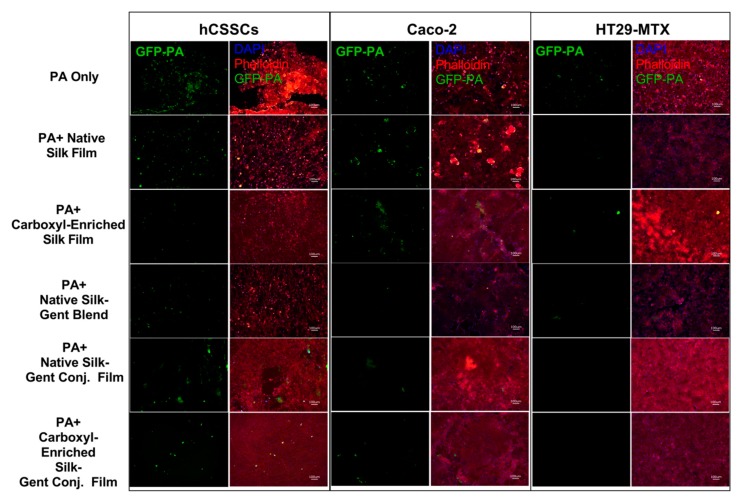
Effects of gentamicin-silk films on *P. aeruginosa* adhesion in stromal (hCSSC) and mucosal (Caco-2 and HT29-MTX) cultures at t = 6 h post-inoculation. Representative IHC images showing relative cell density and bacterial growth within each treatment group.

Therapeutic effectiveness in the context of protection against stromal and mucosal cell loss during infection was determined by applying water-annealed silk films with and without gentamicin blended or conjugated to the surface of hCSSCs, Caco-2, and HT29-MTX cultures followed by GFP-PA inoculation. Post-6 h, *P. aeruginosa* growth was apparent in untreated groups, native silk control, and native silk–gentamicin conjugated samples (Figure 6).

**Figure 7:**
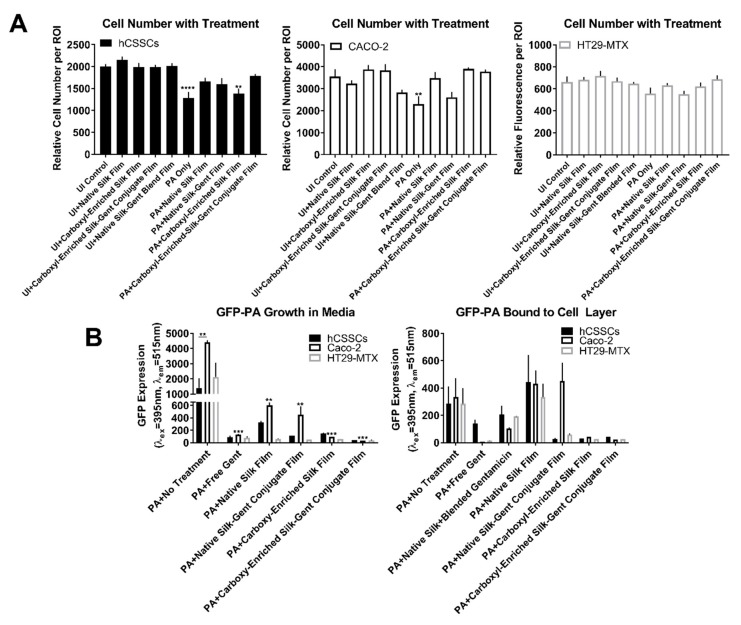
Effects of gentamicin–silk films on stromal (hCSSC) and mucosal (Caco-2 and HT29-MTX) cell retention relative to growth of *P. aeruginosa* (PA) at t = 6 h post-inoculation. (**A**) Quantification of relative cell number per region of interest (ROI) assessed using ImageJ based on the number of nuclei (n = 7–17); (**B**) Abundance of GFP-labelled *P. aeruginosa* (GFP-PA) isolated in the conditioned media and adhered to the cell layer (n = 3). Error bars represent standard error of the mean. Statistical significance shown compared to the PA + No Treatment control for each respective cell type. * *p* ≤ 0.05, ** *p* ≤ 0.01, *** *p* ≤ 0.001, **** *p* ≤ 0.0001 based on a two-way ANOVA.

HT29-MTX cultures showed relatively high cell retention with infection in the untreated PA-infected group, with both Caco-2 cells and hCSSCs showing significant cell loss, correlating with increased GFP-PA abundance (Figure 6 and Figure 7A). Small green particulates were apparent in the hCSSC groups treated with carboxyl-enriched gentamicin conjugated samples and may correlate with silk film debris (Figure 6), which emits green autofluorescence in the absence of GFP-PA (Supplemental Figure S3). We saw little effect on total cell number of hCSSCs, Caco-2, and HT29-MTX cultures as a result of native silk scaffolds in the absence of infection, validating the non-toxic nature of silk biomaterials in biological applications (Figure 7A). Further, we identified protection by native silk scaffolds in terms of cell loss, correlating with lower GFP-PA abundance in the media (*p* ≤ 0.01, Figure 6 and Figure 7B). Likewise, the carboxyl-enriched silk scaffolds exhibited moderate inhibition of GFP-PA growth in the media and cell layer-bound comparable to gentamicin-conjugated and blended films (*p* ≤ 0.001, Figure 7B). The carboxyl-enriched silk films reduced the adherence of GFP-PA to the cell layer in the presence or absence of gentamicin-conjugation, suggesting that the reduced abundance of free bacteria in samples with water-annealed films may be related to bacterial adherence to the scaffolds directly, rather than strictly bactericidal effects (Figure 7B).

## 3. Materials and Methods

### 3.1. PDMS Mold Preparation

Patterned polydimethylsiloxane (PDMS) molds were generated as previously described [42]. Briefly, the base and curing agent (Dow Corning Sylgard 184 Silicone Encapsulant Clear 0.5 kg Kit, Dow Corning, Midland, MI, USA) were mixed in a ratio of 9:1, manually stirred for 1 min, followed by desiccation for 30–60 min under vacuum to remove bubbles. The PDMS mixture was then poured over a micropatterned diffraction grating with microgrooves (Edmund Optics, Barrington, NJ, USA) and allowed to solidify overnight at room temperature.

### 3.2. Silk Film Preparation

Silk fibroin was isolated from cocoons produced by *Bombyx mori* silkworms (Tajima Shoji Co., Yokohama, Japan) according to previously published protocols [23]. Briefly, an aqueous silk solution was obtained by boiling cocoons in 0.02 M sodium bicarbonate (Sigma, St. Louis, MO) for 30 min, followed by drying and dissolution in 9.3 M lithium bromide for 2–4 h at 60 °C. Dialysis of the soluble silk solution (molecular weight cut-off (MWCO) = 3500 Da) against distilled water over three days was performed to obtain a silk solution of ~7% w/v. Silk films were prepared on patterned or non-patterned PDMS molds. A 1% w/v silk solution was applied to the surface of the PDMS mold, allowed to dry for 48 h, and water-annealed (25 mmHg) at room temperature for 2.5 h. To form porous films, 0.05% w/v polyethylene oxide (PEO, Sigma) was included with the 1% w/v silk solution, followed by incubation of set films in distilled water to remove residual PEO forming pores. Ethanol-sterilization prior to use in cell culture was performed by incubating films in 70% v/v ethanol for 15 min at room temperature, followed by washing in phosphate-buffered saline (PBS) and storage at 4 °C. For gentamicin blended films, sterilization was performed using UV-light exposure (overnight at room temperature).

### 3.3. Silk Sponge Preparation

Silk was isolated as a solution of 5–7% w/v in distilled water. Sodium chloride (Sigma) was filtered through stainless steel sieves with aperture size between 500 and 600 nm in diameter (Fisher Scientific, Hampton, NH, 04-884-1AM (600 μM), 04-884-1AN (500 μM)). To generate porous sponges, a 2 mL solution of 5% w/v silk in distilled water was added to a mold of 15 mm diameter. Then, 4 g of sifted sodium chloride was added uniformly to each mold containing the silk solution and allowed to dry for 48 h covered in a chemical hood. The lids were then removed, and the molds placed in distilled water to allow leaching of salt from the silk sponge over three days with frequent water changes. Silk sponges were isolated and cut to 1 mm in height and 6 mm in diameter, followed by autoclave sterilization and storage in Dulbecco’s PBS (DPBS) at 4 °C until further use.

### 3.4. Synthesis of Carboxyl-Enriched Silk

Aqueous silk solutions were diluted to 0.6% w/v, and pH was adjusted to 13 via addition of 10 M sodium hydroxide. Solid chloroacetic acid was added to the solution (final concentration—10 M), and gently stirred at room temperature for 1 h to mediate carboxylation of serine and tyrosine residues on the silk backbone. Monobasic sodium phosphate was added as a buffer (4 mg/mL), and pH was adjusted to ~7 with 10 M hydrochloric acid. The solution was stirred for 30 min, followed by dialysis (MWCO = 20,000 Da) against distilled water for three days. The silk solution was then lyophilized and stored at 4 °C until further use. The concentration of the silk solution was determined based on weight to volume measurements. Further analysis by mass spectrometry and infrared spectroscopy (IR) were performed.

### 3.5. EDC-Mediated Gentamicin–Silk Conjugation

Native or carboxyl-enriched silk was conjugated to gentamicin using EDC coupling. Briefly, 50 mg of silk was dissolved in 5 mL of 0.1 M 2-(N-Morpholino)ethanesulfonic acid (MES) buffer at pH 4.7 and incubated with 0.27 mM EDC with stirring. A 1 mL aliquot of 50 mg/mL gentamicin dissolved in distilled water (Gibco) was added, followed by adjusting pH to 6–7 using 10 M sodium hydroxide and incubation for 5 h at room temperature with gentle stirring. The silk solution was isolated and dialyzed (MWCO = 20,000 Da) against distilled water for three days with six water changes in total. Isolated silk was lyophilized and maintained at 4 °C until further use. Coupling of gentamicin to silk was validated using liquid chromatography-tandem mass spectrometry (LC-MS/MS) based on elution time of gentamicin, parent ion m/z ratio, and the fragmentation pattern compared to a gentamicin standard.

### 3.6. Infrared Spectroscopy

Infrared (IR) spectroscopy was performed on dried films using FTIR Jasco 6200 (Jasco, Tokyo, Japan) with an attenuated total reflectance accessory fitted with a germanium crystal (Pike Tech, Madison, WI, USA) based on previous protocols [39]. Briefly, following background subtraction, spectra were collected using 32 scans (600–4000 cm^−1^) at a resolution of 4 cm^−1^.

### 3.7. Mass Spectrometry

We performed liquid chromatography tandem mass spectrometry (LC-MS/MS) as previously described [35]. Briefly, experiments were performed on an Agilent 1200 series high performance liquid chromatography (HPLC) instrument and Agilent 6410 triple-quadruple mass spectrometer (Agilent Technologies, Santa Clara, CA, USA). Acid hydrolysis was performed on samples and standards prior to analysis (2 M HCl incubation at 60 °C for 1 h), and isolated solids were re-suspended in MS-grade acetonitrile and water (75%:25% v/v) at a concentration of 250 μg/mL. Then, 20 μL samples were injected into a hydrophobic interaction liquid chromatography (HILIC) column (Zorbax HILIC Plus, Agilent Technologies, Santa Clara, CA, USA) under a gradient elution of acetonitrile and water. A multiple reaction monitoring (MRM) method identified tyrosine and gentamicin (m/z transitions 182.1 > 136.1 and 478.4 > 322.2, respectively, collision energy 7). LC and MS spectra were collected for each sample condition and compared to standards.

### 3.8. Ultraviolet (UV)/Visible Spectroscopy

To measure transparency of films, silk films were cast into each well of a 24-well plate and water-annealed at 25 mmHg at room temperature for 2.5 h. Absorbance of light within the visible spectrum (300–800 nm) was measured pre- and post-film application and plotted as % transmittance.

### 3.9. Cell Culture

All protocols were approved by the Institutional Biosafety Committee at Tufts University (registration # 2017-MRIA49) according to institutional and federal guidelines. Experiments were performed in an approved BSL2 laboratory environment.

#### 3.9.1. *Pseudomonas aeruginosa*

GFP-labelled *P. aeruginosa* (GFP-PA) was obtained commercially (ATCC^®^ 10145GFP™, ATCC, Manassas, VA, USA) containing a multicopy vector encoding GFPmut3 expressed under the P_lac_ promoter with ampicillin-resistance. Individual bacterial colonies were generated by streaking the frozen stock onto standard lysogeny broth (LB) agar containing 100 μg/mL ampicillin. Liquid cultures in LB broth containing 300 μg/mL ampicillin were maintained at 37 °C with vigorous shaking (250 rpm) until absorbance measurements reached 0.8 AU at λ_abs_ = 600 nm. Infection studies were performed using bacterial suspensions of 10^4^–10^6^ colony forming units/mL.

#### 3.9.2. Bacterial Abundance

GFP-PA fluorescence was measured spectroscopically from isolated conditioned media and cell lysates using a plate reader (SpectraMax, Molecular Devices, Sunnyvale, CA, USA). Briefly, conditioned media was isolated and suspended in 1% v/v Triton-X-100 in DPBS, followed by fluorescence measurements at λ_ex_ = 395 nm and λ_em_ = 515 nm.

#### 3.9.3. Corneal Stromal Cells

Human corneal stromal stem cells (hCSSCs) isolated from the limbus were kindly provided by Jim Funderburgh (University of Pittsburg), as previously described [43]. hCSSCs were subcultured up to passage 3–5 in proliferation media [low glucose Dulbecco’s Modified Eagle Medium (DMEM, Gibco, Grand Island, NY, USA), MCDB-201 (Sigma, St. Louis, MO, USA), 2% FBS (Gibco), 10 ng/mL platelet-derived growth factor (PDGF), 10 ng/mL epidermal growth factor (EGF), 5 mg/mL insulin-transferrin-selenium (Gibco), 0.1 mM 2-phospho-L-ascorbic acid trisodium salt (Sigma), 10^−8^ M dexamethasone, and 50 μg/mL gentamicin (Life Technologies, Grand Island, NY, USA)]. Upon 90% confluency, cells were incubated in differentiation media (Advanced DMEM, 10 ng/mL fibroblast growth factor-2 (Sigma), 0.1 ng/mL transforming growth factor-β3, 50 μg/mL gentamicin, and 1% antibiotic-antimycotic (Gibco)) for two weeks. Cells were then seeded at concentrations of 1 × 10^5^ cells/cm^2^ and cultured for one week in low serum media (Advanced DMEM (Gibco), 1% FBS, 300 μg/mL ampicillin) until inoculation. Expression of keratocyte markers, keratocan, and lumican was confirmed by immunohistochemistry (Supplemental Figure S4).

#### 3.9.4. Caco-2 and HT29-MTX Cells

Mucosal cell lines (Caco-2 and HT29-MTX) were commercial-sourced from ATCC (Rockville, MD, USA) and Public Health England Culture Collections (Salisbury, England), respectively. Both cell types were cultured in DMEM containing 10% FBS and 1% antibiotic–antimycotic until 90% confluency, seeded at a density of 1 × 10^5^ cells/cm^2^, and maintained in low serum media (Advanced DMEM, 1% FBS, 300 μg/mL ampicillin) for two days until inoculation.

### 3.10. De-Cellularization of Corneal Tissue

Porcine whole eyes were commercially-sourced from Savenor’s Market (Cambridge, MA, USA) and fresh from Animal Technologies (Tyler, TX, USA). Corneal tissue was dissected from the posterior segments of the eye and washed three times in DPBS. De-cellularization was performed with the addition of the ionic detergent, 0.5% w/v sodium dodecyl sulfate (SDS) (Sigma) in PBS, followed by incubation overnight at 4 °C with agitation. The tissue was then washed with DPBS 3X and incubated with DNase and RNase (Qiagen RNeasy Plus Kit, Hilden, Germany) for 3 h at room temperature with rocking to remove cellular DNA and RNA, followed by washing. Tissue was stored in sterile DPBS with 1× antibiotic–antimycotic until inoculation.

### 3.11. Cell Counting

ImageJ (1.52a, National Institutes of Health, Bethesda, MD, USA) was used to analyze IHC images based on the DAPI (4’,6-diamidino-2-phenylindole) signal [44]. Briefly, images taken with a 10× objective lens were converted to an eight-bit tiff and the threshold was set to black and white in ImageJ. Particle analysis was applied using size ranges of 0-infinity and circularity of 0–1 (Supplemental Figure S5). For images of HT29-MTX cultures, total fluorescence was assessed using set thresholds for the DAPI channel with the average integrated density determined for each region of interest.

### 3.12. Viability Assay

Vybrant^®^ MTT cell proliferation assay was utilized to assess cell viability based on the manufacturer’s protocol (Life Technologies, Molecular Probes, Eugene, OR, USA). Briefly, stromal (hCSSCs) and mucosal (Caco-2 and HT29-MTX) cells were cultured in 96-well plates seeded at a cell density of 1 × 10^4^ cells per well; maintained in low serum media without phenol red; and inoculated with GFP-PA for t = 1, 2, 4, and 6 h. At the respective timepoints, media was aspirated, and cells were washed gently with DPBS and re-suspended in 1.2 mM MTT reagent in phenol red-free corneal epithelial media (ATCC) for 2 h at 37 °C, followed by cell lysis using 50 μL dimethyl sulfoxide (DMSO) per well and absorbance measurements at 540 nm.

### 3.13. Immunohistochemistry and Protein Staining

Cultures were isolated and fixed in 4% w/v paraformaldehyde in PBS (Santa Cruz Biotechnology, Dallas, TX, USA) for 15 min at room temperature, washed three times with DPBS, and permeabilized with 0.1% v/v Triton-X-100 (Sigma) in Dulbecco’s phosphate-buffered saline (DPBS) (Gibco) for 15 min at room temperature. Cultures were then blocked with 2.5% w/v bovine serum albumin (Thermofisher, Waltham, MA, USA) in DPBS for 1 h at room temperature with rocking, followed by incubation in the following anti-human primary antibodies overnight at 4 °C or room temperature with rocking: Alexa Fluor™ 647 phalloidin (Rb, 1:40, ThermoFisher, A22287), keratocan (Rb, 1:100, Thermofisher, PA5-58733), and lumican (1:100, ThermoFisher, PA5-14570). The nuclear stain, 4′,6-diamidino-2-phenylindole (DAPI) (0.1 μg/mL in DPBS) (Sigma), was incubated with samples immediately prior to imaging for 5 min at room temperature under static conditions, followed by washing in 0.1% Tween-20 (v/v) (Sigma) in DPBS. FilmTracer™ SYPRO™ Ruby Biofilm Matrix Stain (Molecular Probes, Eugene, OR, USA) was used to stain exogenous protein by incubating 1× solution with bacterial cultures and/or scaffolds for 15 min at room temperature. Excess solution was aspirated, and samples were washed with DPBS for 5 min with rocking (3×).

### 3.14. Fluroescence Microscopy

The Keyence BZ-X700 fluorescent microscope (Keyence, Elmwood Park, NJ, USA) was used to image all samples using 10× or 20× objectives and the following filters: GFP (λ_ex_ = 470 nm and λ_em_ = 525 nm) and Texas Red (λ_ex_ = 560 nm and λ_em_ = 630 nm).

### 3.15. Scanning Electron Microscopy

Silk scaffolds were prepared as described and desiccated under vacuum overnight at room temperature. Dried scaffolds were cut into small pieces (~10 mm × 10 mm) and mounted onto a sample stub using conductive tape. Samples were sputter-coated with gold and imaged by SEM (Hitachi scanning electron microscope, S-4800, Tokyo, Japan).

### 3.16. Statistical Analysis

Data were analyzed using GraphPad Prism 7 for Windows (GraphPad Software, Version 7.03, La Jolla, CA, USA, www.graphpad.com). Statistical significance was determined using an analysis of variance (ANOVA) for grouped analyses with alpha set to 0.05. A *p*-value <0.05 was considered statistically significant.

## 4. Conclusions

This study focused on developing an effective therapeutic against *P. aeruginosa* infections via surface-toxicity of the antibiotic, gentamicin, bound to silk scaffolds. We validated the antimicrobial effects of gentamicin-blended and conjugated biomaterials against acute bacterial infection. Our results revealed that the antimicrobial effects of silk biomaterials containing chemically-bound gentamicin are effective against both planktonic and adhered bacterial cultures of *P. aeruginosa*, supporting the application of silk–gentamicin conjugates as coatings or as porous films to prevent infection. Furthermore, our results support previous work by others [7], which conjugated gentamicin to bovine serum albumin, in showing that immobilization of gentamicin maintains bactericidal effects against *P. aeruginosa* in vitro. The advantages of our findings in this context applies carbodiimide-chemistry to form a covalent bond between gentamicin and the biocompatible silk-based scaffold with tuneable biomechanical properties. Further studies are required to verify that gentamicin-loaded scaffolds inhibit *P. aeruginosa* infection in a physiological environment with the presence of inflammatory cells and tear flow in vivo.

The advantage of this approach is the development of a transparent, stabilized therapeutic for application to inanimate surfaces to reduce the bacterial load available for transmission. We posit that short-term applications of the therapeutic may be applied using non-water-annealed silk–gentamicin scaffolds, allowing spatial and temporal diffusion of the antibiotic into the surrounding environment. Furthermore, in the context of prophylactic use, the application of silk–gentamicin conjugated scaffolds following water-annealing to control β-sheet content within the biomaterial [38,45] may prove useful in preventing bacterial adhesion and growth on devices requiring long-term stability in environmentally exposed settings with a high risk for infection, such as catheters or post-surgical applications. The antimicrobial effects of the silk–gentamicin conjugates support our hypothesis that a silk-based system may be effective as a solution- or coating-based therapeutic to reduce infection. Further studies are warranted to determine the effects of topical application on disruption of tear film perfusion and maintenance in the context of corneal applications, as well as concerns regarding the development of drug resistance in the prophylactic use of antimicrobial lenses [46]. The application of this silk-based system to conjugate other therapeutics, including anti-inflammatory drugs or non-specific antimicrobials (e.g., silver), may be useful alternatives to antibiotic or small-molecule conjugation. Overall, our study reveals a novel approach to develop biocompatible coatings using silk and established EDC-chemistry that can be applied to a number of small molecules or peptides to reduce bacterial infection or growth.

## Supplementary Materials

The following are available online at https://www.mdpi.com/2079-4983/10/3/41/s1. Figure S1: Relative transparency of native silk films; Figure S2: Morphology and growth patterns of GFP-labelled *P. aeruginosa* (GFP-PA) in cultures; Figure S3: Fluorescent microscopy images of uninfected scaffolds stained with Sypro Ruby; Figure S4: Expression of keratocyte markers (keratocan and lumican) by uninfected human corneal stromal stem cells (hCSSCs); Figure S5: Representative images of total nuclei within each region of interest (ROI) in stromal (hCSSCs) and mucosal cells (Caco-2 and HT29-MTX) following 6 h post-inoculation with *P. aeruginosa*.
