# Supplementary material for: Silk-Based Therapeutics Targeting Pseudomonas aeruginosa
Source: J Funct Biomater. 2019 Sep 13;10(3):41. doi: 10.3390/jfb10030041 (PMC6787730; doi:10.3390/jfb10030041)
Supplement: Supplementary file 1 [file jfb-10-00041-s001.pdf]

Journal of *Functional Biomaterials*

Supplemental Materials

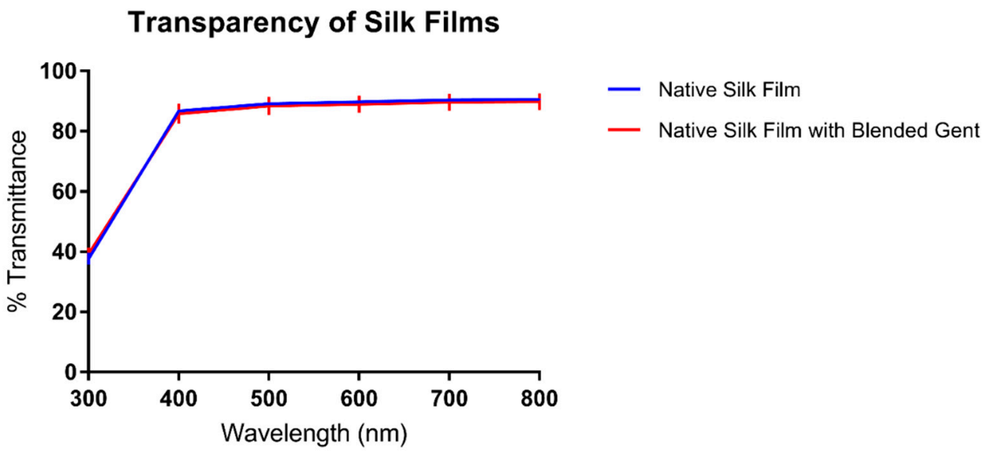

Figure S1. Relative transparency of native silk films measured spectroscopically based on absorbance within the wavelength range of 300–800 nm. Error bars represent standard deviation with n = 3.

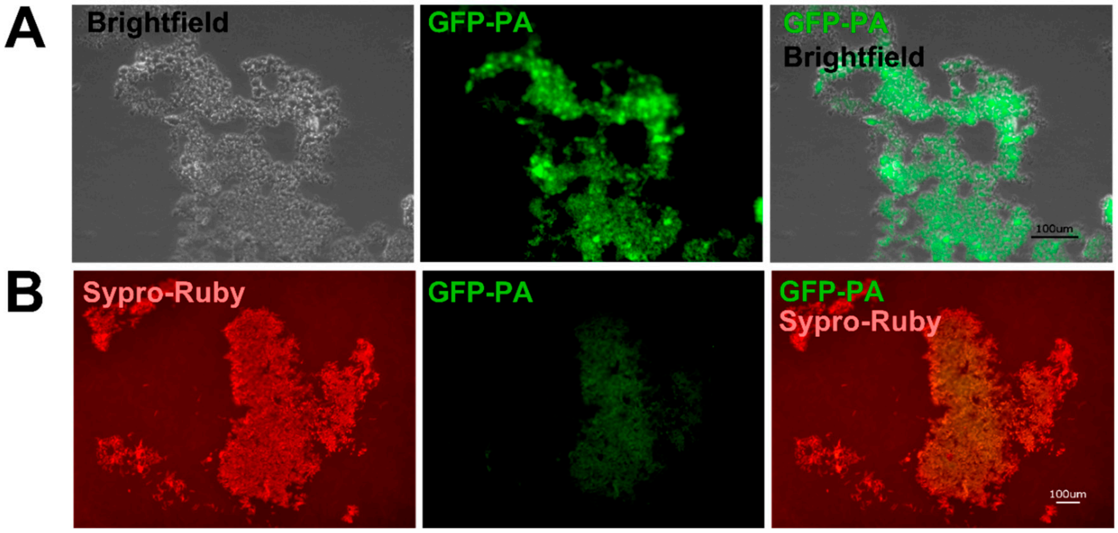

Figure S2. Morphology and growth patterns of GFP-labelled *P. aeruginosa* (GFP-PA) in cultures. (**A**) GFP-PA cultured on conventional tissue culture plastic for t = 24 h and imaged using λ_{ex} = 470 nm and λ_{em} = 525 nm; (**B**) Growth of GFP-PA on a de-cellularized porcine cornea for t = 48 h. Images taken on fixed tissue samples using Sypro-Ruby (λ_{ex} = 560 nm and λ_{em} = 630 nm) and GFP-PA immunofluorescence.

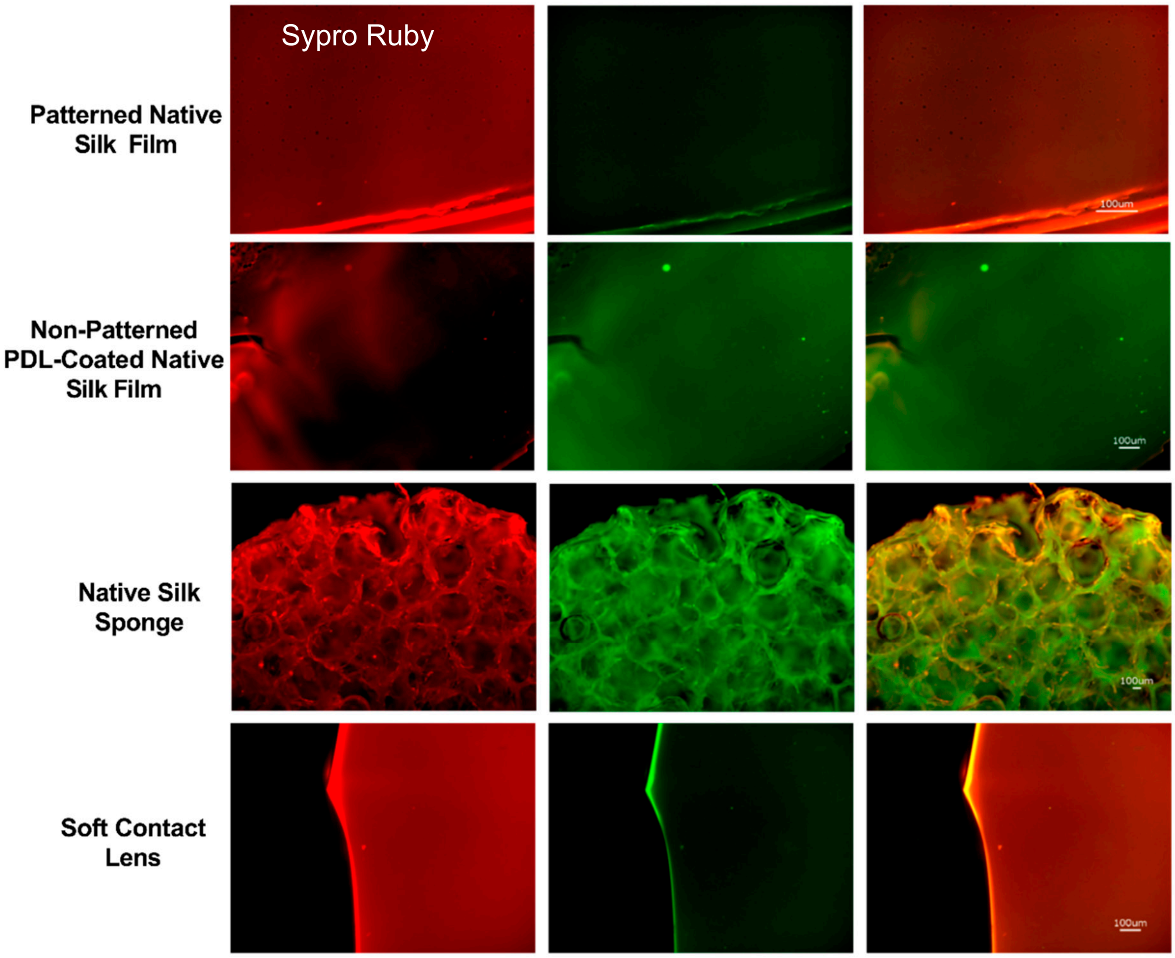

Figure S3. Fluorescent microscopy images of uninfected scaffolds stained with Sypro Ruby. The green channel (λ_{ex} = 470 nm and λ_{em} = 525 nm) shows high green autofluorescence from silk scaffolds.

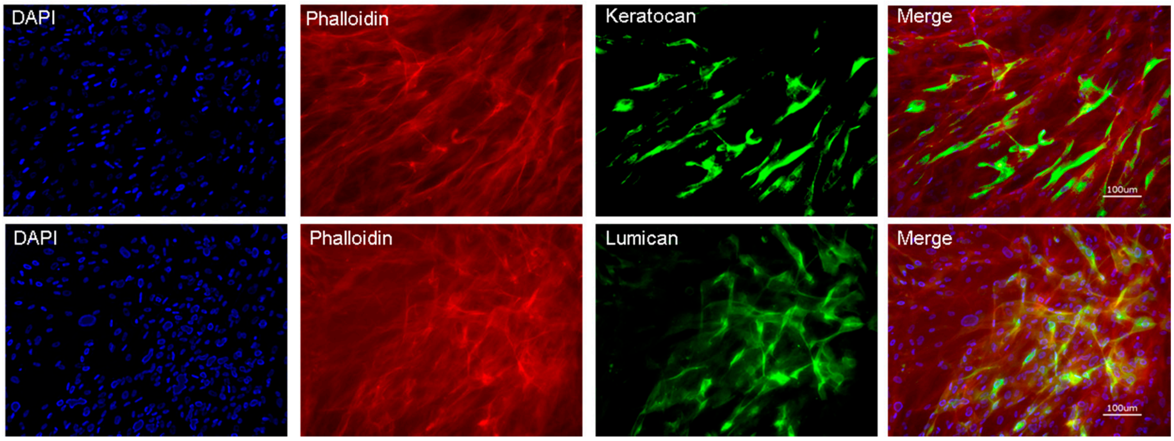

Figure S4. Expression of keratocyte markers (keratocan and lumican) by uninfected human corneal stromal stem cells (hCSSCs).

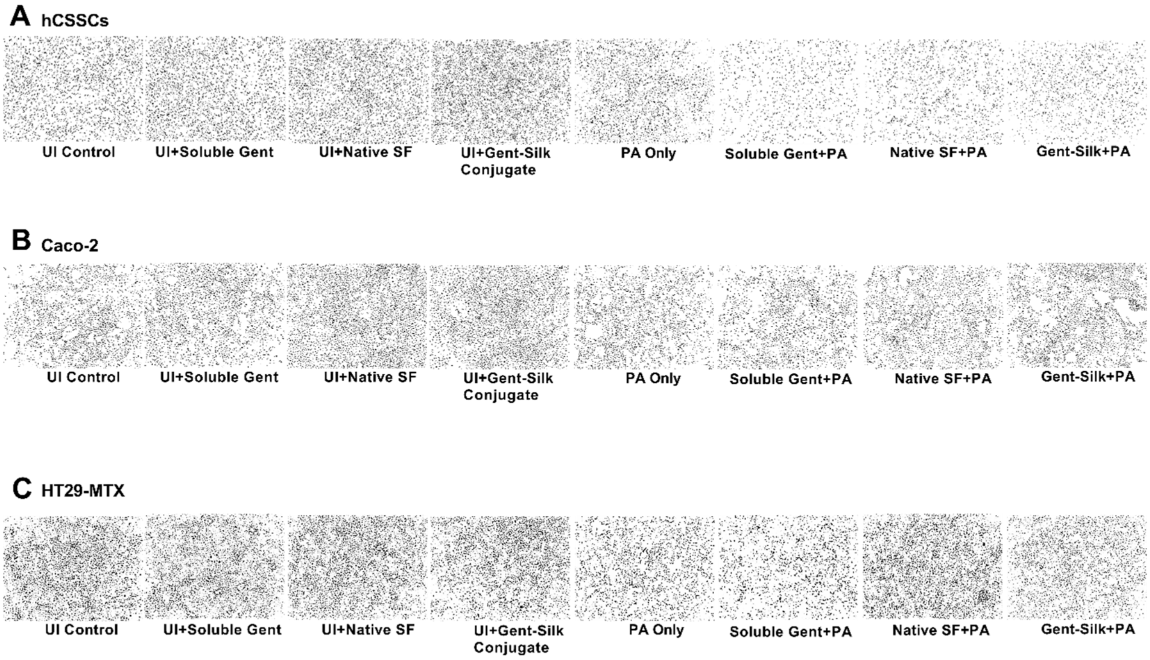

Figure S5. Representative images of total nuclei within each region of interest (ROI) in stromal (hCSSCs) and mucosal cells (Caco-2 and HT29-MTX) following 6 h post-inoculation with *P. aeruginosa*. ImageJ particle analysis used to determine relative cell number for (**A**) hCSSCs and (**B**) Caco-2 cells (**C**) with total fluorescence analysis based on the DAPI channel used to estimate relative cell number for HT29-MTX cells.
